# Back pain in elementary schoolchildren is related to screen habits

**DOI:** 10.3934/publichealth.2020045

**Published:** 2020-07-29

**Authors:** Erivelton Fernandes França, Michel Monteiro Macedo, Fernando Francisco Pazello Mafra, Gabrielle Mitico Miyake, Romildo Torres da Silva, Tania Regina de França, Thyago Ribeiro dos Santos, João Pedro da Silva Junior, Victor Keihan Rodrigues Matsudo, Nelson Morini Junior, Eduardo Natali Della Valentina, Fábio Dupart Nascimento, Rodrigo Álvaro Brandão Lopes Martins

**Affiliations:** 1 Núcleo de Pesquisas Tecnológicas (NPT) da Universidade de Mogi das Cruzes (UMC), Mogi das Cruzes, SP, Brazil; 2 Centro Universitário Carlos Drummond de Andrade (UNIDRUMMOND), São Paulo, SP, Brazil; 3 Universidade de São Paulo (USP), São Paulo, SP, Brazil; 4 Centro de Estudos do Laboratório de Aptidão Física de São Caetano do Sul (CELAFISCS), São Caetano, SP, Brazil; 5 Universidade do Vale do Paraíba (UNIVAP), São José dos Campos, SP, Brazil

**Keywords:** back pain, screen habits, childhood physical activity, child obesity

## Abstract

**Purpose:**

Verify the influences of physical activity level, nutritional status and screen habits on the prevalence of back pain in Brazilian students.

**Methods:**

The sample consisted of 577 schoolchildren (female = 274; male = 303) aged between 10 and 16 years old, regularly enrolled in the 6^th^ grade of elementary school living in the metropolitan area of the Alto Tietê of the state of São Paulo, Brazil. The prevalence, intensity and frequency of pain was verified with the Back Pain Assessment Instrument. The usual practice of physical activity was verified with the Physical Activity Questionnaire for Older Children/Adolescent. Nutritional status was analyzed using Body Mass Index. Screen habits were obtained through a previously structured questionnaire.

**Results:**

The Chi-square test indicated that pain complaint and its prevalence in the cervical region are significantly higher in females (p < 0.05). The multiple logistic regression test revealed that watching television influences the prevalence of cervical pain and that the use of more than one screen increases the occurrence of low back pain in male students (p < 0.05).

**Conclusion:**

Female students were the most affected by back pain complain, especially in the cervical region. However, factors associated with the prevalence of back pain were found only in males.

## 1. Introduction

The scientific literature suggests that the prevalence of back pain is influenced by multiple factors such as behavioral, physical, psychosocial and genetic [1]. Thus, age, gender, sleep habits, physical activity level, anxiety and depression, heredity, and excessive screen time can be predictors of back pain [1]–[8]. Regrettably, back pain affects a representative portion of the world's adult population, especially in developing countries such as Brazil [9]. A cross-sectional study conducted with data from the 2013 National Health Survey, conducted by the Brazilian Institute of Geography and Statistics in partnership with the Ministry of Health, brought interesting epidemiological data. This research was applied to 63.348 adults from private households in both urban and rural areas, in five geographic macro-regions, 27 federative states their capitals. These results pointed out that, in Brazil, chronic back pain was reported by 18.5% of adults, 15.5% in men and 21.1% in women [10]. Chronic back pain in the highly productive working age group impacted negatively on the country's economy [11]. In addition, it should be noted that this condition may also negatively affect the ability to perform activities of daily living [12].

In addition to the adult population, studies indicate that children and adolescents have also been affected by back pain [13]. Complaining of back pain is a serious public health problem that affects a large part of the world's population in the most varied age groups [1]–[3], including schoolchildren and adolescents. In a Danish study conducted by Skoffer [14], which evaluated 546 schoolchildren aged 14–17, it was found that 60% of students complained of low back pain in the last 12 months. Martinez-Crespo and collaborators [15], in investigating the prevalence of back pain in 887 schoolchildren in the City of Seville, Spain, found an even higher prevalence, in this case, 66% in the last year. In Brazilian students a similar phenomenon is observed. Noll and collaborators found that in 1720 schoolchildren aged 11 to 16 years living in the city of Teutonia, Rio Grande do Sul, 55% reported having had back pain in the last 3 months [16].

Although the prevalence of back pain is well described in the literature [1],[5]–[8], the factors involved in its genesis are not yet fully understood and, in some cases, are conflicting, especially in schoolchildren. It is also worth mentioning that most studies are limited to describing the prevalence of back pain in schoolchildren, leaving gaps about the frequency and intensity of reported pain, and often limited to only spine region. Thus, the aim of this study was to describe the frequency and intensity of back pain reported in the cervical, thoracic and lumbar regions, as well as to verify the influence of physical activity level, nutritional status and screen habits (habits regarding the use of technological resources that have an electronic display such as cell phone, television, video game, computer and tablet) on the prevalence of pain, in Brazilian schoolchildren residing in the metropolitan region of Alto Tietê in São Paulo state.

## 2. Method and materials

### 2.1. Study design and ethics approval

The present research is a descriptive, cross-sectional study with primary data collection and sample for convenience. Data were collected during August and December 2017, during classes in the afternoon between 1 pm and 6 pm, in five state public schools in the cities of Itaquaquecetuba and Mogi das Cruzes, belonging to the metropolitan region of Alto Tietê in the state of São Paulo, Brazil. The sample of the present study consisted of 577 schoolchildren of both sexes (female = 274, male = 303) aged 10 to 16 years, regularly enrolled in the 6^th^ grade of elementary school. All procedures adopted in the research were approved by the Institutional Review Board of the University of Mogi das Cruzes, (protocol: 2.459.458).

### 2.2. Inclusion criteria

Only schoolchildren whose legal guardians signed the Informed Consent Form and who expressed the desire to participate in data collection were included in the study. In addition, to have the data considered in this research, it was necessary to be literate and not having physical and/or neurological disabilities that would prevent them from normally performing daily physical activities and using electronic devices such as television, cell phone, video game, tablet and computers.

### 2.3. Procedures

The prevalence, intensity and frequency of pain was determined with the Back Pain Assessment Instrument (BPI) [17] showing good reproducibility and applicability in Brazilian adolescents [18]. The BPI is a questionnaire with multiple choice closed questions that enables the identification of pain in areas present in the posterior region of the body, including the cervical, thoracic and lumbar areas of the spine. With the application of the BPI, it was possible to obtain information regarding the region of the back pain, the intensity and its frequency, as well as to verify if the individuals complained of pain during the questionnaire application.

The habitual physical activity level was verified through the Physical Activity Questionnaire for Older Children (PAQ-C), which was designed for children aged 8 to 13 years and Physical Activity Questionnaire for Adolescent (PAQ-A) for adolescents between the ages of 14 and 18 years [19]. Based on the answers to the questionnaires, it was possible to classify the students in (“Very Sedentary”, “Sedentary” = Physically inactive), (“Moderately Active”, “Active” and “Very Active” = Physically active).

Body weight and height were obtained using an anthropometric digital scale with a coupled stadiometer (Toledo Prix A 2098 PP), duly calibrated, with an accuracy of 100 grams and 0.1 centimeters, respectively. The body mass index (BMI) was calculated by dividing the body weight (kg) by the square value of the height (m), and its final value expressed in (kg/m^2^). In this way the students were classified as thinner, eutrophic, overweight or obese [20].

Screen habits (SH) were verified through a questionnaire previously structured by the researchers and it was composed of 10 closed and open questions (4 multiple choice questions and 6 essay questions). After being duly completed, it was possible to identify the most used types of screen, the time of use and also, the purpose that the students use of the technological resources described. In our study, we considered excessive screen time more than 2 hours a day [21], according to the recommendations of the American Academy of Pediatrics [21].

### 2.4. Data analysis

The normality of the data was verified by the Kolmogorov-Smirnov test. Height and level of physical activity showed normal distribution and were compared using the parametric student's t-test. However, age, weight, BMI and screen time did not present a normal distribution, being then compared using the Mann-Whitney non-parametric test. Prevalence, intensity and frequency of back pain were compared between the sexes using the test of Chi-square with correction of Yates, when necessary. Factors associated with cervical, thoracic and lumbar spine pain were investigated with the multiple logistic regression test. The results were considered statistically significant only when p < 0.05. All analyzes were performed using Bioestat version 5.3 statistical software.

## 3. Results

Table 1 showed the values of age, mass, height, BMI, daily TT and level of physical activity (PAQ-C score). The student's t-test revealed a difference between genders in relation to height and level of physical activity, the same was pointed out by the Mann-Whitney U test in relation to the variables of mass and BMI. In summary, male students presented lower height, mass and BMI, but a higher level of physical activity when compared to female schoolchildren (p ≤ 0.05).

**Table 1.** Characteristics of subjects.

Continuous variables	Male (n = 303)	Female (n = 274)	p-value
	Mean (SD)	Mean (SD)	
Age (Years)	11.95 (0.65)	12.02 (0.74)	(U_(39321.00)_; p = 0.27)
Weight (Kg)	43.64 (11.80)	47.20 (11.35)	(U_(32738.50)_; p = 0.00)*
Height (m)	1.51 (0.07)	1.53 (0.07)	(T_(-2.9146)_; p = 0.00)*
BMI (Kg/m^2^)	18.89 (4.12)	18.89 (4.12)	(U_(33513.50)_; p = 0.00)*
Daily ST (h)	3.72 (1.65)	3.92 (1.65)	(U_(36869.50)_; p = 0.14)
Score PAQ-C	2.70 (0.71)	2.45(0.72)	(T_(3.9088)_; p = 0.00)*

Note: n = Number of students; SD = Standard Deviation; ST = Screen time; h = Hours; Kg = Kilograms; BMI = Body Mass Index; m = Meters; m^2^ = Height in meters squared; Score PAQ-C = Indicates in arbitrary unit the level of average physical activity predicted by the Physical Activity Questionnaire for Children; p-value = value of significance; * = p ≤ 0.05 according student's T test or Mann-Whitney U test; # ST (♂ n = 296), and (♀ n = 268); # PAQ-C (♂ n = 251).

Approximately 25% of students complained of back pain at the exact time of data collection, and the prevalence of this event was significantly higher in female students compared to male students (33.58% vs. 17.80%, p ≤ 0.01). Among the regions investigated, the one with the most complaints of pain by the students was cervical, around 30%, which, as well as the previously described variable (pain complaint at the time of data collection), was significantly higher in females compared to gender male (36.86% vs. 25.00%, p ≤ 0.05). Regarding the number of regions with pain reports (1, 2 or 3), we observed that most students complained of pain in a single region, almost 28%. This variable was significantly higher in female students compared to male students (22.94% vs. 34.32%) (Table 2).

**Table 2.** Pain Profile in schoolchildren.

Categorical variables	Male (n = 292)	Female (n = 268)	p-value
	n (%)	n (%)	
Pain complaint	123 (42.12)	161 (60.07)	(χ^2^_(5.534)_; p = 0.01)*
Pain at the moment	52 (17.80)	90 (33.58)	(χ^2^_(10.310)_; p = 0.00)*
Cervical pain	73 (25.00)	101 (36.86)	(χ^2^_(5.113)_; p = 0.02)*
Thoracic spine pain	69 (23.63)	87 (31.75)	(χ^2^_(2.747)_; p = 0.09)
Low back pain	45 (15.41)	62 (22.62)	(χ^2^_(3.264)_; p = 0.07)
1 region of the spine	67 (22.94)	92 (34.32)	(χ^2^_(4.096)_; p = 0.04)*
2 regions of the spine	38 (13.01)	43 (16.04)	(χ^2^_(0.580)_; p = 0.44)
3 regions of the spine	13 (4.45)	22 (8.20)	(χ^2^_(2.397)_; p = 0.12)

Cervical pain	Male (n = 73)	Female (n = 101)	p-value
	n (%)	n (%)	

Cervical pain mild	44 (60.27)	47 (46.53)	(χ^2^_(0.749)_; p = 0.38)
Cervical pain medium	22 (30.13)	34 (33.66)	(χ^2^_(0.039)_; p = 0.84)
Cervical pain Strong	5 (6.84)	16 (15.84)	(χ^2^_(1.870)_; p = 0.17)
Cervical pain Unbearable	2 (2.73)	4 (3.96)	(χ^2^_(0.000)_; p = 1.00)
7x per week	1 (1.36)	7 (6.93)	(χ^2^_(1.665)_; p = 0.19)
4–6 x per week	5 (6.84)	6 (5.94)	(χ^2^_(0.008)_; p = 0.93)
1–3x per week	20 (27.39)	30 (29.70)	(χ^2^_(0.007)_; p = 0.93)
1–4x per month	21 (28.76)	31 (30.69)	(χ^2^_(0.002)_; p = 0.96)
1–4x per year	26 (35.61)	27 (26.73)	(χ^2^_(0.570)_; p = 0.45)

Thoracic spine pain	Male (n = 69)	Female (n = 87)	p-value
	n (%)	n (%)	

Thoracic spine pain mild	38 (55.07)	47 (54.02)	(χ^2^_(0.004)_; p = 0.94)
Thoracic spine pain medium	18 (26.08)	22 (25.28)	(χ^2^_(0.008)_; p = 0.92)
Thoracic spine pain Strong	9 (13.04)	14 (16.09)	(χ^2^_(0.055)_; p = 0.81)
Thoracic spine pain unbearable	4 (5.79)	4 (4.59)	(χ^2^_(0.002)_; p = 0.96)
7x per week	4 (5.79)	3 (3.44)	(χ^2^_(1.583)_; p = 0.20)
4–6 x per week	6 (8.69)	4 (4.59)	(χ^2^_(0.414)_; p = 0.51)
1–3x per week	21 (30.43)	27 (31.03)	(χ^2^_(0.012)_; p = 0.91)
1–4x per month	24 (34.78)	33 (37.93)	(χ^2^_(0.015)_; p = 0.90)
1–4x per year	14 (20.28)	20 (22.98)	(χ^2^_(0.018)_; p = 0.89)

Low back pain	Male (n=45)	Female (n=62)	p-value
	n (%)	n (%)	

Low back pain mild	29 (64.44)	29 (46.77)	(χ^2^_(0.665)_; p = 0.41)
Low back pain medium	8 (17.77)	21 (33.87)	(χ^2^_(1.446)_; p = 0.22)
Low back pain Strong	5 (11.11)	7 (11.29)	(χ^2^_(0.080)_; p = 0.77)
Low back pain unbearable	3 (6.66)	5 (8.06)	(χ^2^_(0.014)_; p = 0.90)
7x per week	1 (2.22)	2 (3.22)	(χ^2^_(0.085)_; p = 0.77)
4–6 x per week	4 (8.88)	4 (6.45)	(χ^2^_(0.005)_; p = 0.94)
1–3x per week	12 (26.66)	20 (32.25)	(χ^2^_(0.065)_; p = 0.79)
1–4x per month	17 (37.77)	24 (38.70)	(χ^2^_(0.015)_; p = 0.90)
1–4x per year	11 (24.44)	12 (19.35)	(χ^2^_(0.076)_; p = 0.78)

Note: n = Number of students; p-value = value of significance; * = p ≤ 0.05 according χ^2^ Chi-square test; (%) = Gender-proportional sampling value.

**Table 3.** Cervical pain and associated factors.

Categorical variables (cases)	Male (n = 73)			Female (n = 101)		
	P	OR	CI 95%	p	OR	CI 95%
Nutritional status:						
Not overweight: (Male = 49; Female = 67)						
Overweight: (Male = 24; Female = 34)	0.27	1.46	0.74–2.90	0.78	0.91	0.48–1.74
Physical activity level:						
Physically active: (Male = 51; Female = 79)						
Physically inactive: (Male = 22; Female = 22)	0.99	1.00	0.51–1.95	0.76	0.89	0.45–1.79
Screen time:						
≤two hours: (Male = 17; Female = 18)						
≥two hours: (Male = 56; Female = 83)	0.93	0.96	0.44–2.10	0.19	0.58	0.26–1.30
Cellphone:						
No: (Male = 33; Female = 85)						
Yes: (Male = 40; Female = 16)	0.16	0.17	0.01–2.05	0.95	0.93	0.08–11.20
Television:						
No: (Male = 48; Female = 85)						
Yes: (Male =2 5; Female = 16)	0.04*	0.07	0.01–0.98	0.87	0.81	0.06–10.49
Computer:						
No: (Male = 59; Female = 97)						
Yes: (Male = 14; Female = 4)	0.62	0.53	0.04–6.88	0.90	0.83	0.05–13.93
Video Game:						
No: (Male = 68; Female = 100)						
Yes: (Male = 5; Female = 1)	0.17	0.15	0.01–2.29	0.94	120163.70	0.00–Infinity
Tablet:						
No: (Male = 66; Female = 99)						
Yes: (Male = 7; Female = 2)	0.58	0.47	0.03–6.85	0.85	0.72	0.02–23.72
More than one electronic device:						
No: (Male = 6; Female = 13)						
Yes: (Male = 67; Female = 88)	0.41	0.65	0.23–1.81	0.10	2.57	0.83–7.92
Entertainment:						
No: (Male = 7; Female = 20)						
Yes: (Male = 66; Female = 81)	0.91	19350.07	0.00–Infinity	0.18	0.17	0.01–2.26
Communication:						
No: (Male = 6; Female = 29)						
Yes: (Male = 67; Female = 72)	0.92	7066.97	0.00–Infinity	0.19	0.18	0.01–2.45
School activities:						
No: (Male = 72; Female = 101)						
Yes: (Male = 1; Female = 0)	0.99	0.68	0.00–Infinity	0.91	0.00	0.00–Infinity

Note: OR = Odds Ratio; CI = Confidence Interval; * = p ≤ 0.05.

**Table 4.** Thoracic spine pain and associated factors.

Categorical variables (cases)	Male (n = 69)			Female (n = 87)		
	P	OR	CI 95%	p	OR	CI 95%
Nutritional status:						
Not overweight: (Male = 55; Female = 58)						
Overweight: (Male = 14; Female = 29)	0.07	0.49	0.23–1.09	0.63	0.85	0.44–1.64
Physical activity level:						
Physically active: (Male = 26; Female = 16)						
Physically inactive: (Male = 43; Female = 71)	0.45	1.27	0.67–2.45	0.55	0.80	0.40–1.64
Screen time:						
≤two hours: (Male = 19; Female = 9)						
≥two hours: (Male = 50; Female = 78)	0.22	0.63	0.31–1.32	0.18	1.80	0.75–4.33
Cellphone:						
No: (Male = 32; Female = 19)						
Yes: (Male = 37; Female = 68)	0.92	11896.58	0.00–Infinity	0.91	29770.46	0.00–Infinity
Television:						
No: (Male = 54; Female = 74)						
Yes: (Male = 15; Female = 13)	0.91	19994.20	0.00–Infinity	0.91	34903.44	0.00–Infinity
Computer:						
No: (Male = 59; Female = 83)						
Yes: (Male = 10; Female = 4)	0.91	27398.27	0.00–Infinity	0.91	30448.01	0.00–Infinity
Video game:						
No: (Male = 68; Female = 87)						
Yes: (Male = 1; Female = 0)	0.93	2567.45	0.00–Infinity	0.99	0.72	0.00–Infinity
Tablet:						
No: (Male = 63; Female = 86)						
Yes: (Male = 6; Female = 1)	0.91	32065.66	0.00–Infinity	0.91	35288.55	0.00–Infinity
More than one electronic device:						
No: (Male = 6; Female = 11)						
Yes: (Male = 63; Female = 76)	0.73	1.19	0.42–3.42	0.89	1.07	0.37–3.15
Entertainment:						
No: (Male = 11; Female = 31)						
Yes: (Male = 58; Female = 56)	0.41	0.33	0.03–4.56	0.91	31102.84	0.00-Infinity
Communication:						
No: (Male = 59; Female = 56)						
Yes: (Male = 10; Female = 31)	0.52	0.41	0.03–6.15	0.90	51141.99	0.00–Infinity
School activities:						
No: (Male = 68; Female = 87)						
Yes: (Male = 1; Female = 0)	0.90	0.00	0.00–Infinity	0.99	0.93	0.00–Infinity

Note: OR = Odds Ratio; CI = Confidence Interval; * = p ≤ 0.05.

**Table 5.** Low back pain and associated factors.

Categorical Variables (cases)	Male (n = 45)			Female (n = 62)		
	P	OR	CI 95%	p	OR	CI 95%
Nutritional status:						
Not overweight: (Male = 33; Female = 44)						
Overweight: (Male = 12; Female = 18)	0.64	0.80	0.33–2.00	0.22	0.62	0.28–1.35
Physical activity level:						
Physically active: (Male = 15; Female = 18)						
Physically inactive: (Male = 30; Female = 44)	0.78	1.11	0.51–2.47	0.15	1.71	0.81–3.64
Screen time:						
≤two hours: (Male = 11; Female = 10)						
≥two hours: (Male = 34; Female = 52)	0.45	0.70	0.29–1.75	0.68	0.82	0.32–2.11
Cellphone:						
No: (Male = 27; Female = 52)						
Yes: (Male = 18; Female = 10)	0.19	0.19	0.02–2.37	0.94	0.91	0.07–11.61
Television:						
No: (Male = 37; Female = 58)						
Yes: (Male = 8; Female = 7)	0.18	0.16	0.01–2.35	0.49	0.38	0.03–5.72
Computer:						
No: (Male = 37; Female = 61)						
Yes: (Male = 8; Female = 1)	0.46	0.37	0.03–5.25	0.43	0.27	0.01–6.97
Video game:						
No: (Male =4 3; Female = 62)						
Yes: (Male = 2; Female = 0)	0.10	0.07	0.00–1.77	0.95	0.00	0.00–Infinity
Tablet:						
No: (Male = 45; Female = 61)						
Yes: (Male = 0; Female = 1)	0.15	0.09	0.00–2.45	0.90	0.00	0.00–Infinity
More than one electronic device:						
No: (Male = 4; Female = 8)						
Yes: (Male = 41; Female = 54)	0.03*	0.32	0.12–0.91	0.66	0.77	0.25–2.43
Entertainment:						
No: (Male = 10; Female = 24)						
Yes: (Male = 35; Female = 38)	0.17	0.16	0.01–2.18	0.69	0.59	0.04–7.93
Communication:						
No: (Male = 37; Female = 39)						
Yes: (Male = 8; Female = 23)	0.42	0.33	0.02–4.86	0.95	0.92	0.07–12.68
School activities:						
No: (Male = 43; Female = 62)						
Yes: (Male = 2; Female = 0)	0.64	0.42	0.01–15.91	0.93	0.00	0.00–Infinity

Note: OR = Odds Ratio; CI = Confidence Interval; * = p ≤ 0.05.

From the answers provided to the BPI we conclude that, in the 3 regions investigated (cervical, thoracic and lumbar), mild pain, approximately 54%, was the most reported. Finally, there was not difference between male and female groups for the possible pain classifications (mild, medium, strong and unbearable) (p > 0.05) (Table 2).

In the cervical region the frequency of most reported pain was 1–4 times a year (around 31%). In the thoracic region, the frequency of pain most reported by schoolchildren was 1–4 times a month (almost 36%), as was the lumbar region (approximately 38%). Regarding the possible frequency ratings suggested by the IDC (7x per week, 4–6x per week, 1–3x per week, 1–4x per month, 1–4x per year), we found no significant difference between sexes (p > 0.05) (Table 2).

The use of television influenced by 7% the prevalence of cervical pain in male students (OR = 0.0716; p = 0.0480) (Table 3) and that the use of more than one screen increased the occurrence of low back pain by 32% (OR = 0.3272; p = 0.0329) (Table 5). For the other variables no significant association was observed (p > 0.05).

## 4. Discussion

Swain and collaborators [22] estimated the prevalence of back pain in adolescents from 28 countries aged 9 to 17 years. It was observed that the average worldwide prevalence of back pain in adolescents was 37%, with Poland and the Czech Republic having the lowest and highest rates (27.7% vs. 50.5%), respectively. More recently, in a systematic review study conducted by Calvo-Muñoz and collaborators [23] analyzed the risk factors for low back pain in 137.877 individuals (between 9 and 16 years) from 5 continents, it was observed that prevalence of low back pain varied between 15.25% and 38.98% in the world population. Despite this, no consistent data were found, in this population, on the factors associated with the prevalence of pain. These results reinforces the complexity of the factors involved in the genesis of back pain in childhood. Corroborating these studies, we also found a high prevalence of pain complaints in our students reporting back pain at least once in the last year (around 51%) and more than 25% complained of pain during the test application. It is noteworthy that, in both cases, female students pain complaints were significantly higher when compared to male students (60.07% vs. 42.12%). Similar results were obtained in a longitudinal study conducted by Noll and collaborators in Brazilian schoolchildren [24]. Similarly they found a higher prevalence of back pain in female students. It is possible to speculate, therefore, that hormonal and biochemical mechanisms may interfere negatively in the greater pain reported by female students [25]. Moreover, this points to a complexity involved in the genesis of back pain that, despite being well described, is multifactorial, and maybe influenced by sex [1]–[8].

Our study did not merely describe the prevalence of back pain in schoolchildren, nor did we restrict ourselves to investigating this phenomenon in just one region of the spine, as is usually the case [26]. Using BPI, it was possible to obtain relevant information about this phenomenon. Regarding the pain site, we found that the cervical region is the most affected (30.93%) being more prevalent in females, followed by the thoracic (27.69%) and lumbar (19.01%) regions, which was similar between males and females. Another interesting fact is that among the students who reported back pain, 20.85% of them complain of pain in more than one spinal region. Taken together, these results point to the need for future research with schoolchildren investigating back pain complaints in all spinal regions (cervical, thoracic and lumbar) and not just the lower back, as many studies have prioritized [3],[5],[12],[27],[28].

Two other interesting points were the frequency and prevalence of back pain for each region of the spine. For the cervical region the frequency of most reported pain was 1–4 times a year (approximately 31%). In the thoracic region, the frequency of pain most reported by schoolchildren was 1–4 times a month (almost 36%), as was the lumbar region (around 38%). Regarding the possible frequency ratings suggested by the BPI (7x per week, 4–6x per week, 1–3x per week, 1–4x per month, 1–4x per year), there was no significant difference between genders for any of the regions investigated (p > 0.05). Regarding the frequency of back pain reported by schoolchildren, some studies that also investigated this variable deserve to be pointed out. Skoffer [14] found in Danish schoolchildren aged 14–17 that 60% of students have complained of back pain in the last 12 months. Martinez-Crespo and collaborators [15] found an even higher prevalence (66%) of schoolchildren in Spain. Another highly representative study conducted by Noll and collaborators[16] found that in 1720 Brazilian schoolchildren aged 11 to 16 years living in the city of Teutonia, Rio Grande do Sul, 55% reported having had back pain in the last 3 months. These studies points out to variations in the frequency of back pain reported by schoolchildren. Thus, future studies should also focus on multiple factors that may interfere with the increase of this frequency.

In an attempt to elucidate the multiple factors associated with the etiology of back pain in schoolchildren, we analyzed the influence of physical activity level, nutritional status and screen habits on the prevalence of pain in the thoracic and lumbar cervical regions. Among the variables analyzed, we found that the use of television influences cervical pain in 7% in male students and that students who used more than one technological resource (television, computer, video game, tablet or cell phone) in their habits daily, where 32% were more prone to low back pain. This is likely to be due to the excessive amount of time students sit in positions (sitting or lying down) that overload the spine [29]. It is also worth remembering that besides the leisure time, students are most of the time seated in chairs and tables in classrooms almost always disregarding their anthropometric characteristics, thus making school furnitures inappropriate for use [30]. If preventive measures are not taken, back pain may negatively influence the quality of life of children and adolescents [31], including school performance [32]. It is therefore crucial that new research and preventive interventions such as Postural Education programs [33],[34] be carried out, especially in adolescence, an extremely critical period in the intensification of back pain. In addition, if left untreated during childhood, back pain may last into adulthood [22].

Our study has some limitations. For example, although this study has a moderate sample size, in this case, 577 (female = 274, male = 303), it was a cross-sectional study with a convenience sample. Thus, the results we obtained do not allow generalization to the entire population of Brazilian schoolchildren. In addition, our study did not consider other important variables that may also be associated with the prevalence of back pain, such as posture when sitting down during classes, weight of the backpack in which students transported school materials and socioeconomic factors. Lastly, some logistic regression results showed large confidence interval, which indicate a large error of estimation. Hence, the readers should interpret these results with cautions.

## 5. Conclusion

Female students were the most affected by back pain complain, especially in the cervical region. Only in males, we identified factors associated with the prevalence of back pain as it was the negative association television cervical pain the use of more than one screen predisposing to the occurrence of low back pain.
